# Brain specific Lamellipodin knockout results in hyperactivity and increased anxiety of mice

**DOI:** 10.1038/s41598-017-05043-3

**Published:** 2017-07-14

**Authors:** Cristian Bodo, Cathy Fernandes, Matthias Krause

**Affiliations:** 1 King’s College London, Randall Division of Cell and Molecular Biophysics, New Hunt’s House, Guy’s Campus, London, SE1 1UL UK; 2 King’s College London, Institute of Psychiatry, Psychology & Neuroscience, Social, Genetic & Developmental Psychiatry Centre (MRC), 16 De Crespigny Park, Denmark Hill, London, SE5 8AF UK

## Abstract

Lamellipodin (Lpd) functions as an important signalling integrator downstream of growth factor and axon guidance receptors. Mechanistically, Lpd promotes actin polymerization by interacting with F-actin and the actin effectors Ena/VASP proteins and the SCAR/WAVE complex. Thereby, Lpd supports lamellipodia protrusion, cell migration and endocytosis. In the mammalian central nervous system, Lpd contributes to neuronal morphogenesis, neuronal migration during development and its *C*. *elegans* orthologue MIG-10 also supports synaptogenesis. However, the consequences of loss of Lpd in the CNS on behaviour are unknown. In our current study, we crossed our Lpd conditional knockout mice with a mouse line expressing Cre under the CNS specific Nestin promoter to restrict the genetic ablation of Lpd to the central nervous system. Detailed behavioural analysis of the resulting Nestin-Cre-Lpd knockout mouse line revealed a specific behavioural phenotype characterised by hyperactivity and increased anxiety.

## Introduction

Lamellipodin (Lpd; official HGNC gene symbol: RAPH1) is a vertebrate member of the MRL (MIG-10, RIAM, Lamellipodin) protein family which also includes RIAM in vertebrates, MIG-10 in *C*. *elegans* and Pico in *Drosophila* ^1–5^. Lpd localises to the very edge of lamellipodia, the tips of filopodia, and to clathrin coated pits^1, 6^. At these locations, Lpd functions as an important signalling integrator downstream of growth factor and axon guidance receptors. It is a direct Ras and Rac effector and also functions downstream of PI3-kinase signalling via its Ras-association and PH domains^1, 7–9^. Mechanistically, Lpd recruits Ena/VASP proteins and interacts with endophilin, the SCAR/WAVE complex and F-actin via its proline-rich C-terminal region to promote actin polymerization. Thereby, Lpd supports axonal morphogenesis, endocytosis, lamellipodia protrusion, cell migration and metastasis^6, 7, 9–13^. The Scar/WAVE complex regulates actin nucleation through the Arp2/3 complex and Ena/VASP proteins promote actin filament elongation^14, 15^. Consistently, its orthologue in *C*. *elegans*, MIG-10, functions as a regulator of axon guidance, synaptic vesicle clustering during synaptogenesis and neuronal migration in *C*. *elegans* ^4, 16–18^.

Several lines of evidence indicate that Lpd plays a role in the development of the mammalian CNS. Lpd is expressed uniformly throughout the cortex during embryonic development^19^. Stimulation of primary cortical neurons with the axon guidance cue netrin-1 results in Abl-mediated tyrosine phosphorylation of Lpd. Depletion of Lpd impairs axonal elongation and branching of primary hippocampal neurons. Consistently, Lpd and c-Abl co-overexpression promotes hippocampal axonal morphogenesis in an Ena/VASP protein dependent manner^9^. Moreover, Lpd is required for the M-Ras mediated outgrowth and branching of cortical dendrites, both *in vitro* and *in vivo* ^20^. Depletion of Lpd in pyramidal neurons arising from the ventricular zone during corticogenesis in the mouse embryo elicited an aberrant cortical phenotype, with the cortical neurons switching from a radial to a tangential pattern of migration that leads to their accumulation within the subventricular and intermediate zones^19^. Lpd knockdown also reduced the number of primary processes in multipolar neurons accumulating in the multipolar cell accumulation zone before they initiate their radial migration in the developing cortex^21^.

In summary, the available evidence suggests that Lpd promotes neuronal morphogenesis, neuronal migration during CNS development in mice and synaptogenesis in *C*. *elegans*. Therefore, we set out to evaluate the consequences of loss of Lpd in the CNS on the behaviour of mice by testing them through a comprehensive behavioural screen. We previously had generated conditional knockout mice for Lpd, which we crossed to PGK-Cre general deleter mice on a pure C57BL6 background. Most of these mice died just after birth with the few surviving mice displaying a ventral pigmentation defect. Crossing the Lpd conditional mice to general deleter beta-Actin-Cre mice on a mixed genetic background allowed more offspring to survive. The surviving mice displayed a ventral pigmentation defect which was due to a melanoblast migration/proliferation defect^7^. In our current study, we crossed our Lpd conditional mice with a mouse line expressing Cre under the CNS specific Nestin promoter to restrict the genetic ablation to the central nervous system. Our results revealed a specific behavioural phenotype resulting from the genetic ablation characterized by hyperactivity and increased anxiety in the open field test.

## Results

### Generation of CNS-specific knockout mice for Lpd

**Figure 1:**
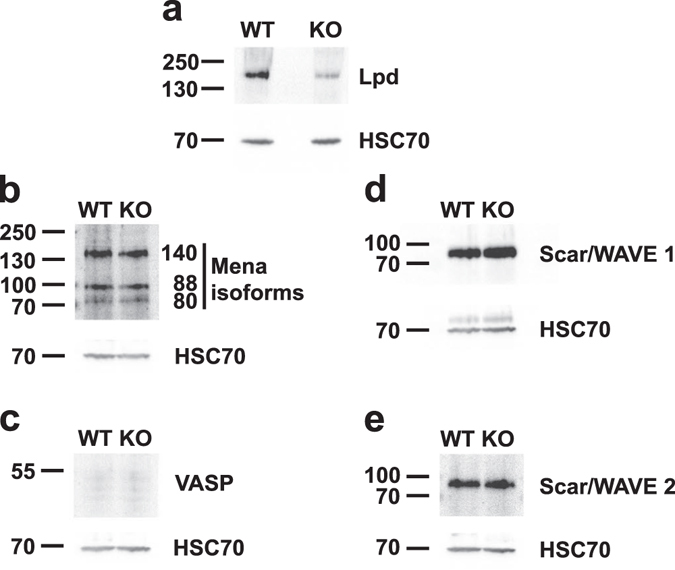
Expression levels of Lpd and associated actin regulators in Nestin-Cre-Lpd KO mice. Equal amounts of protein extracts (30 µg) of the cortices of adult Nestin-Cre-Lpd knockout (KO) and Lpd flox/flox control mice (WT) were separated by SDS-PAGE and analysed by Western blotting with antibodies against (**a**) Lpd, (**b**) Mena (note that three isoforms of Mena of 80, 88, and 140 kDa are expressed in adult brain^33^), (**c**) VASP (note that VASP is not expressed in adult brain^33^), (**d**) Scar/WAVE1, and (**e**) Scar/WAVE2. HSC70 re-probe of each blot serves as the loading control (note that the upper faint band in the HSC70 blot in (**d**) represents the remaining Scar/WAVE1 band. Molecular weight markers are indicated in kDa.

Conditional Lpd knockout mice^7^ were crossed with another line in which Cre was constitutively expressed under the regulation of the nestin promoter to generate Nestin-Cre-Lpd KO mice. From crosses of Lpd flox/flox males with Lpd flox/flox; Nestin-Cre/- females we obtained the expected Mendelian ratio of cre-expressing knockout mice at P10 (47.7% based on 15 individual litters, Z-score = 0.24), indicating that the genetic ablation did not affect viability. In brains of Nestin-Cre-Lpd KO mice we observed loss of Lpd expression and no change in expression levels of the actin effector proteins, Mena, VASP, Scar/WAVE1, and Scar/WAVE2, all of which normally interact with Lpd, when compared to Lpd flox/flox control brains (Fig. 1a–e). The knockout mice exhibited a normal lifespan and no morphological defects were evident. Statistical analysis of adult weight values yielded the expected significant difference between male and female mice (27.90 ± 0.53 grams, SEM, n = 21 vs. 24.11 ± 0.30 grams, SEM, n = 18, F[1,35] = 33.46, p < 0.0001), but no significant effect of genotype (F[1,35] = 0.12, p = 0.7357) and no interaction between the two factors (F[1,35] = 0.46, p = 0.5007, Two-way ANOVA) were observed.

### Basic behavioural phenotype assessment

**Figure 2:**
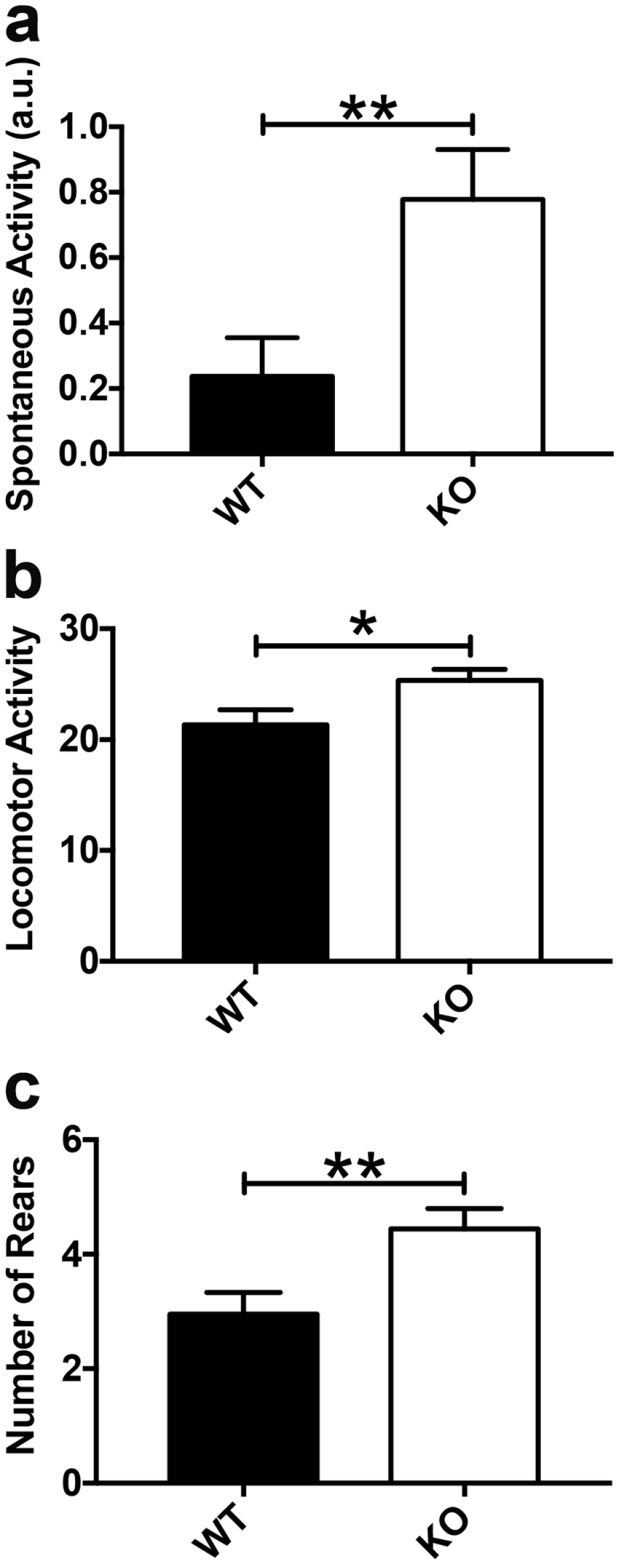
Nestin-Cre-Lpd KO mice exhibited a hyperactive phenotype during the SHIRPA test. (**a**) Spontaneous activity (arbitrary units: 0 = lowest activity; 4 = highest activity) exhibited by the experimental mice during the SHIRPA Test. Nestin-Cre-Lpd KO mice were 3.3 times more active (KO: 0.78 ± 0.15, SEM, n = 18 vs. WT: 0.24 ± 0.12, SEM, n = 21, not normally distributed, two-tailed Mann-Whitney U test, p = 0.0056). (**b**) Mean locomotor activity (defined as total number of squares entered during a 30 s test) recorded during the SHIRPA test. Nestin-Cre-Lpd KO mice (25.33 ± 1.00, SEM, n = 18) showed significantly higher values for this variable compared to their wild type littermates (21.33 ± 1.35, SEM, n = 21; F[1,35] = 5.00, p = 0.0318), with no significant effect of sex (F[1,35] = 0.01, p = 0.9445) or significant interaction between the two factors (F[1,35] = 0.03, p = 0.8588), Two-way ANOVA. (**c**) Average number of rears during the arena test discriminated by genotype. Nestin-Cre-Lpd KO mice showed significantly higher values (KO: 4.44 ± 0.35, SEM, n = 18, vs WT: 2.95 ± 0.38, SEM, n = 20, F[1,34] = 8.04, p = 0.0076), with no significant effect of sex (F[1,34] = 0.85, p = 0.3641) or significant interaction between the two factors (F[1,34] = 0.11, p = 0.7420), Two-way ANOVA, one outlier was removed from the male WT group (11 rears) using the Grubbs method (alpha = 0.05), Statistical significance: *represents p < 0.05; **represents p < 0.01.

Nestin-Cre-Lpd KO (Lpd flox/flox; Nestin-Cre/-) mice and their wild type (Lpd flox/flox) littermates were subjected to a battery of observational tests specifically designed for basic phenotype assessment known as SHIRPA^22^. The levels of spontaneous activity exhibited by Nestin-Cre-Lpd KO mice in the viewing jar were increased 3.3 times compared to those from their wild type littermates (Fig. 2a).

Locomotor activity following transfer to the arena was significantly higher for Nestin-Cre-Lpd KO mice compared to wild type littermates (Fig. 2b), as was the average number of rears they made (Fig. 2c). For both variables, there was no significant effect of sex and no interaction between the sex and the genotype of the mice.

No significant effects of either sex or genotype were observed for the other variables quantified during the SHIRPA assessment.

### Light/Dark box test

**Figure 3:**
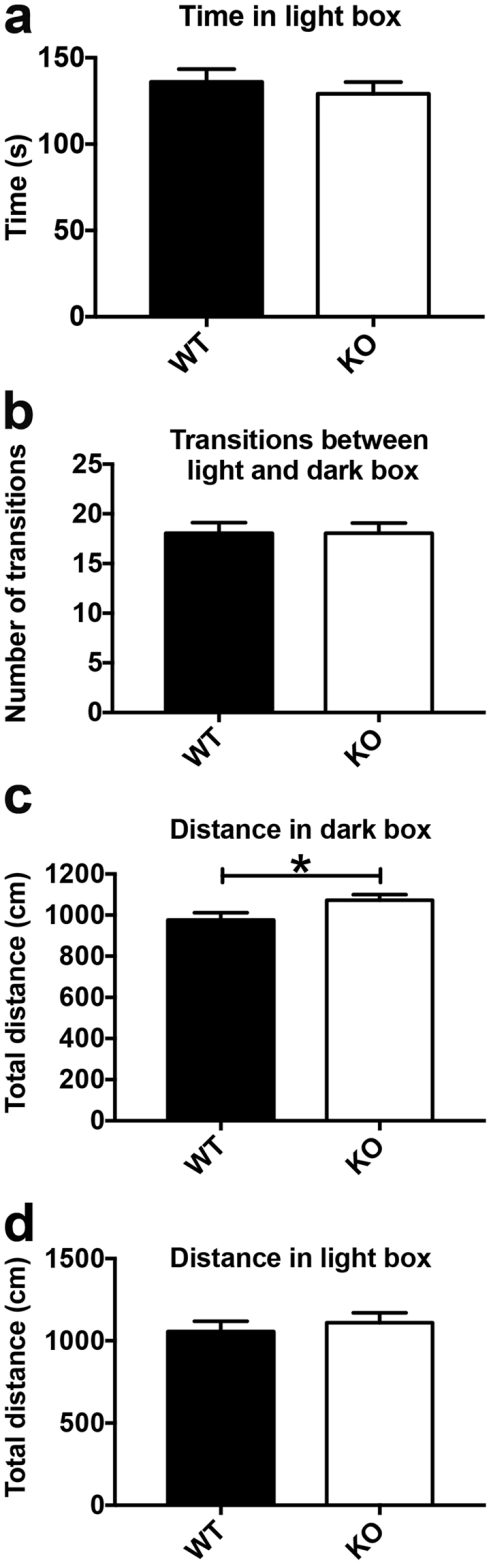
Nestin-Cre-Lpd KO mice displayed normal levels of anxiety in the Light/Dark box but were mildly hyperactive. (**a**) Total time (seconds) spent in the light compartment of the Light/Dark box (WT: 136.00 ± 7.37 s, SEM, n = 20, KO: 129.20 ± 6.76 s, SEM, n = 19, F[1,35] = 0.40, p = 0.5306, Two-way ANOVA). (**b**) Number of transitions between light and dark box (WT: 18.05 ± 1.06, SEM, n = 20, KO: 18.05 ± 1.02, SEM, n = 19, F[1,35] = 0.001, p = 0.9725). (**c**,**d**) Total distance (centimeters) covered by mice while in the dark and light compartment of the Light/Dark box, respectively. Nestin-Cre-Lpd KO mice covered significantly more distance (1072.28 ± 27.41 cm, SEM, n = 19) than their wild type littermates while exploring the dark compartment (976.55 ± 35.34 cm, SEM, n = 20, F[1,35] = 4.41, p = 0.0431) with no significant effect of sex (F[1,35] = 0.32, p = 0.5758) and no interaction between the factors (F[1,35] = 1.23, p = 0.2758, Two-way ANOVA) but no differences were observed in the exploration of the light compartment (Interaction: F[1,35] = 0.2697, p = 0.6068; sex: F[11,35] = 1.23, p = 0.2756; genotype: F[1,35] = 0.49, p = 0.4880, Two-way ANOVA). Statistical significance: *represents p < 0.05.

No significant differences were found in the time the mice spent in the illuminated chamber during the tests or for the number of transitions between the light and the dark box, regardless of their sex or genotype (Fig. 3a and b). However, the total distance covered in the darkened chamber was significantly higher in Nestin-Cre-Lpd KO mice than in wild type individuals (Fig. 3c), with no significant effect of sex. There was no significant effect of either of these factors on the total distance covered in the illuminated chamber (Fig. 3d) or on any of the other variables recorded during this test.

### Open field test

**Figure 4:**
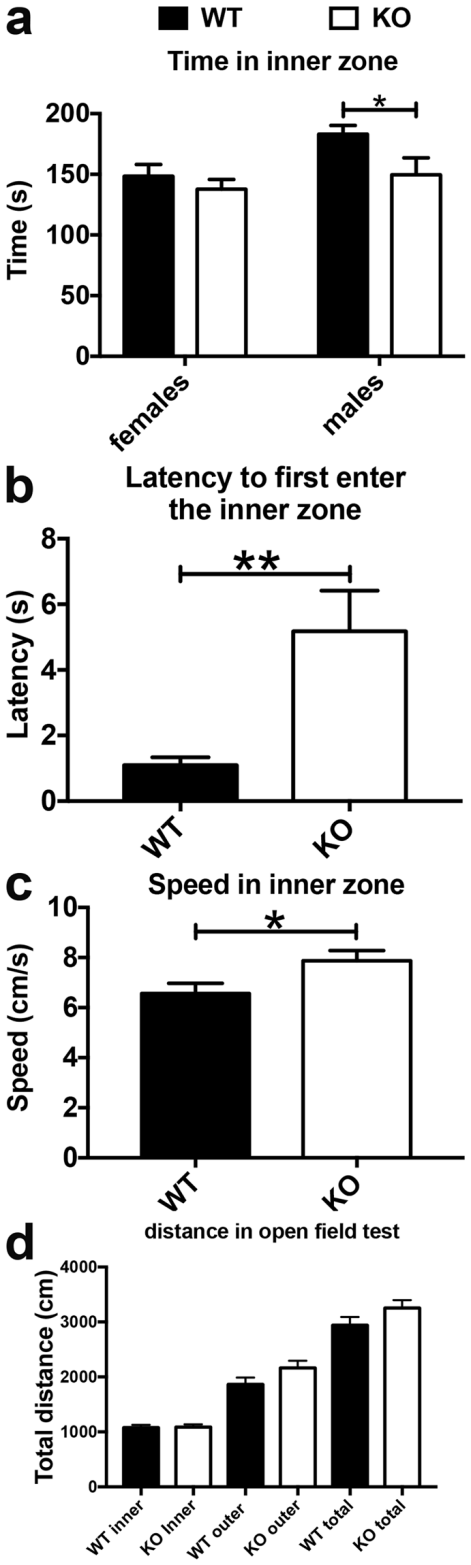
Nestin-Cre-Lpd KO mice exhibited anxiety-like behaviour while exploring the inner zone during the open field test. (**a**) Total time (seconds) spent exploring the virtual inner zone, discriminated by genotype (F[1,35] = 5.09, p = 0.0304) and sex (F[1,35] = 5.62, p = 0.0234) of the experimental mice, respectively. There was no significant interaction between these two factors (F[1,35] = 1.37, p = 0.2499, Two-way ANOVA). Male Nestin-Cre-Lpd KO mice spent significantly less time (149.56 ± 14.01 s, SEM, n = 9) in the inner zone compared to their wild type littermates (183.05 ± 7.23 s, SEM, n = 12, two-tailed, unpaired t-test, t_19_ = 2.28, p = 0.0342). Female Nestin-Cre-Lpd KO mice spent (137.80 ± 7.94 s, SEM, n = 10) in the inner zone compared to their wild type littermates (148.40 ± 9.59 s, SEM, n = 8, two-tailed, unpaired t-test, t_16_ = 0.09, p = 0.4024). (**b**) Latency (seconds) to first enter the inner zone after the mouse was introduced into the test box. The average latency of the Nestin-Cre-Lpd KO mice to first enter the inner zone (5.18 ± 1.24 s, SEM, n = 19) was approximately 4.7 times higher than in wild type individuals (1.10 ± 0.24 s, SEM, n = 19, one outlier was removed from the male WT group (7.6 s) using the Grubbs method (alpha = 0.05), F[1,34] = 9.75, p = 0.0036). There was no significant effect of sex (F[1,34] = 0.01, p = 0.9083) and no interaction between the factors (F[1,34] = 0.09, p = 0.7639, Two-way ANOVA). (**c**) Nestin-Cre-Lpd KO mice were significantly faster in the inner zone (WT: 6.57 ± 0.40 cm/s, SEM, n = 20; KO: 7.87 ± 0.41 cm/s, SEM, n = 19; F[1,35] = 4.28, p = 0.0460) and no differences in the outer zone (F[1,35] = 1.95, p = 0.1714). These variables did not exhibit any significant effect of sex (inner zone: F[1,35] = 1.30, p = 0.2627; outer zone (F[1,35] = 0.11, p = 0.7438) and no interactions between the factors (inner zone: F[1,35] = 0.06, p = 0.8025; outer zone (F[1,35] = 0.27, p = 0.6034, Two-way ANOVA). (**d**) The overall distance moved (in cm) is not significantly different between WT and Nestin-Cre-Lpd KO mice, WT inner vs KO inner, adjusted p = > 0.9999, WT outer vs KO outer, adjusted p = 0.4335, WT total vs KO total, adjusted p = 0.3904, One-way ANOVA, Tukey’s. Statistical significance: *represents p < 0.05; **represents p < 0.01.

In the open field, the time spent exploring the inner zone was influenced by the sex of the mice. Male but not female Nestin-Cre-Lpd KO mice spent significantly less time exploring the inner zone than their wild type littermates (Fig. 4a). There was no significant interaction between these two factors.

For the latency to first enter the inner zone of the arena there was no significant effect of sex. Nestin-Cre-Lpd KO mice showed a significantly delayed exploration of the inner zone of the arena. The average latency to first enter the inner zone was approximately 4.7 times higher than in wild type individuals (Fig. 4b).

The average speed exhibited during exploration of the outer zone of the arena was similar between the genotypes. However, Nestin-Cre-Lpd KO mice were significantly faster when they explored the inner zone compared to wild type littermates (Fig. 4c). The difference between the means represented an approximate increase of 20%. This variable did not exhibit any significant effect of sex. The distance moved in the inner and outer zones, as well as the overall distance traversed during the entire duration of the test, were not significantly different between WT and Nestin-Cre-Lpd KO mice (Fig. 4d).

**Figure 5:**
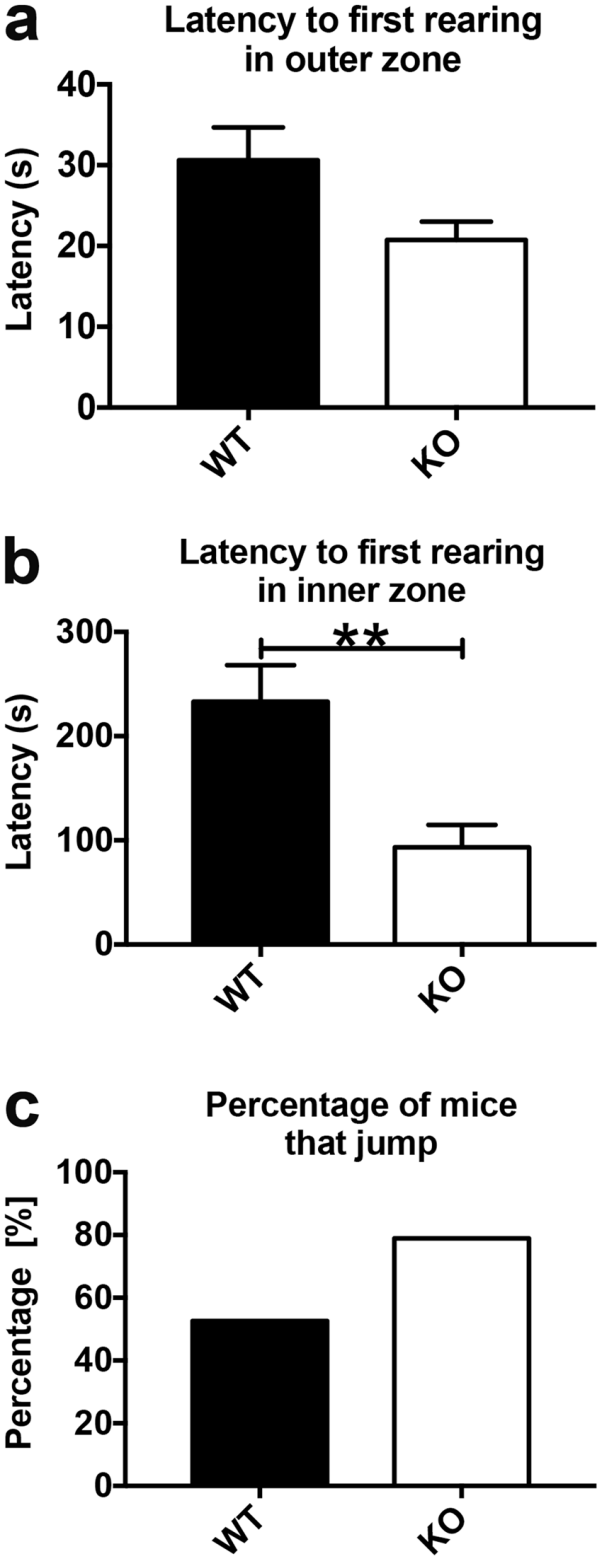
Nestin-Cre-Lpd KO mice exhibited anxiety-like behaviour as well as hyperactivity during the open field test. (**a**,**b**) Latency (seconds) to first rearing in the outer and inner zones. Nestin-Cre-Lpd KO mice exhibited a trend towards lower latencies in the outer zone (**a**) (WT: 30.60 ± 4.09 s, SEM, n = 20, vs. KO: 20.76 ± 2.26 s, SEM, n = 19) that failed to reach significance (F[1,35] = 3.93, p = 0.0555) with no effect of sex (F[1,35] = 0.08, p = 0.7780) nor interaction between the factors (F[1,35] = 0.05, p = 0.8300, Two-way ANOVA). (**b**) However, Nestin-Cre-Lpd KO mice took significantly less time to exhibit the first rearing episode within the inner zone (WT: 233.10 ± 35.06 s, SEM, n = 20 vs. KO: 93.36 ± 21.54 s, SEM, n = 17; F[1,33] = 12.69, p = 0.0011, one outlier was removed from the female KO group (600 s) and one from the male KO group (600 s) using the Grubbs method (alpha = 0.1)). There was no significant effect of sex for this variable (F[1,33] = 2.28, p = 0.1406) nor an interaction between the factors (F[1,33] = 1.09, p = 0.3031, Two-way ANOVA). (**c**) Percentage of experimental mice that exhibited jumping behaviour at any point during the test, discriminated by genotype. 10 out of 20 WT mice and 15 out of 19 KO mice displayed jumping behaviour. The difference between these percentages came close but failed to reach statistical significance (z = 1.91, p = 0.057), chi-square test, Statistical significance: *represents p < 0.05.

The latency to first rear (defined as those instances in which the mouse stood on its hind legs) within the outer zone showed a trend towards lower values in KO mice that just failed to reach significance (Fig. 5a). However, the latency for the first rear within the inner zone was significantly lower for KO individuals than for their wild type littermates (Fig. 5b). There was no significant effect of sex for this variable.

The percentage of experimental mice that exhibited jumping behaviour during the trial was 77.77% for Nestin-Cre-Lpd KO mice and 47.37% for wild type littermates. The difference between these percentages came close but failed to reach statistical significance (Fig. 5c).

### Home cage monitoring

There were no significant effects of either genotype or sex for any of the variables (distance, velocity, heading, turn angle, angular velocity, meander) monitored during the recordings. The average distance covered and speed by each Nestin-Cre-Lpd KO mouse during the recording period was 1599.64 ± 222.90 cm (SEM) and 2.68 ± 0.37 cm/s (SEM) compared to their wild type littermates with 1455.69 ± 244.20 cm (SEM) and 2.41 ± 0.44 cm/s (SEM).

### Novel object exploration

**Figure 6:**
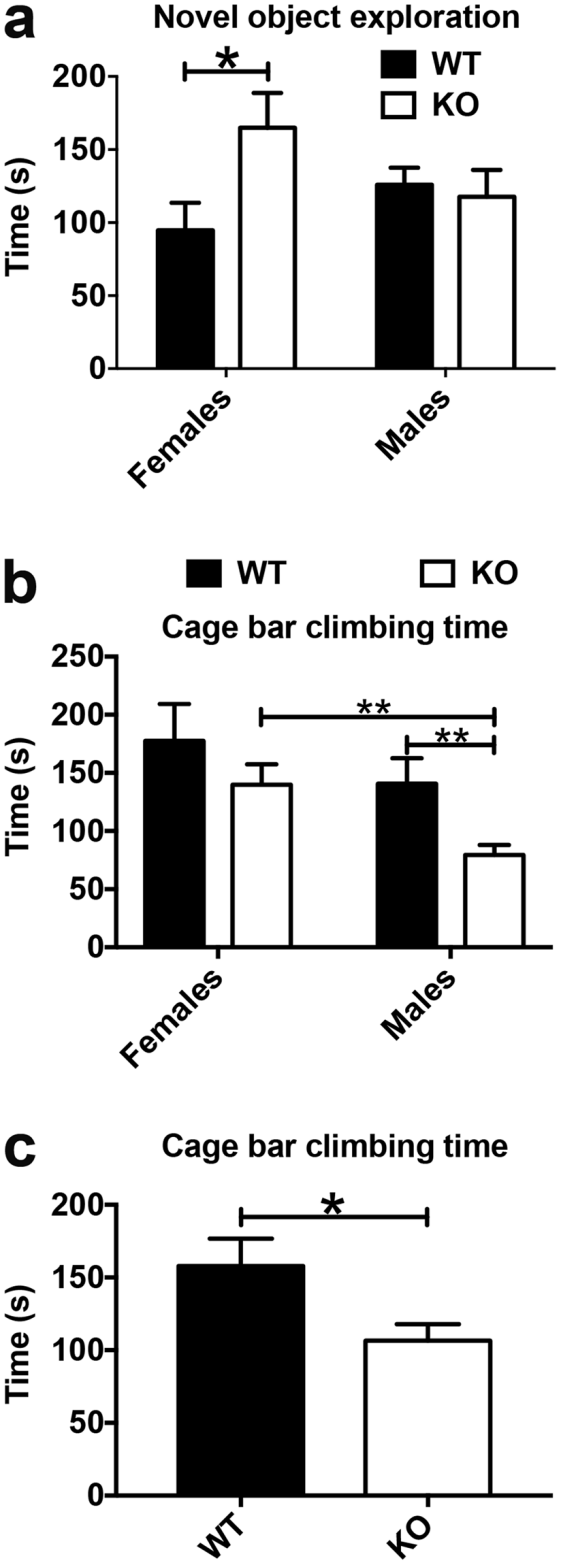
Nestin-Cre-Lpd KO female mice displayed enhanced exploratory behaviour during the novel object exploration test. (**a**) Total time (seconds) spent in active exploration of the novel object during the test, discriminated by sex of the experimental mice (F[1,31] = 4.25, p = 0.0477, Two-way ANOVA). Female Nestin-Cre-Lpd KO mice exhibited significantly higher values for this variable compared to their wild type female littermates (WT: 94.71 ± 18.95 s, SEM, n = 7, vs. KO: 166.70 ± 23.39 s, SEM, n = 9; two-tailed, unpaired t-test, t_14_ = 2.29, p = 0.0381), but there was no difference in the case of male experimental mice (t_17_ = 0.34, p = 0.7378). There were no significant differences between the sexes for either KO mice (two-tailed, unpaired t-test, t_18_ = 1.667, p = 0.1127) or wild type mice (t_13_ = 1.437, p = 0.1744). (**b**) A significant effect of genotype (F[1,31] = 6.53, p = 0.0158) and of sex (F[1,31] = 6.30, p = 0.0175) was detected when the values for total bar climbing time were analysed, with no significant interaction between the two factors (F[1,31] = 0.37, p = 0.5467, Two-way ANOVA). Male Nestin-Cre-Lpd KO mice spent significantly less time climbing the bars than male wild type mice (WT: 140.80 ± 21.85 s, SEM, n = 8 vs KO: 79.45 ± 8.84 s, SEM, n = 11, two-tailed, unpaired t-test, t_17_ = 2.92, p = 0.0096. In addition, female KO mice (139.90 ± 17.47 s, SEM, n = 9) were more prone to engage in this behaviour compared to male KO mice (79.45 ± 8.55 s, SEM, n = 11, two-tailed, unpaired t-test, t_18_ = 3.29, p = 0.0040). (**c**) Total time (seconds) spent climbing the bars of the experimental cage during the test, discriminated by genotype. Wild type individuals were more likely to engage in this behaviour compared to Nestin-Cre-Lpd KO littermates (WT: 157.90 ± 18.79 s, SEM, n = 15 vs KO: 106.70 ± 11.25 s, SEM, n = 20, two-tailed, unpaired t-test, t_33_ = 2.47, p = 0.019). Statistical significance: *represents p < 0.05; **represents p < 0.01.

There was a significant interaction between genotype and sex when the values for total time exploring the novel object were analysed. Nestin-Cre-Lpd KO females explored the novel object for a significantly longer time compared to wild type females (Fig. 6a), whereas no significant differences were detected in the case of male mice. There were no significant differences between the sexes for either KO mice or wild type mice.

A significant effect of genotype and of sex was detected when the values for total bar climbing time were analysed, with no significant interaction between the two factors. Female KO mice were more prone to engage in this behaviour compared to male KO mice. In addition, male Nestin-Cre-Lpd KO mice spent significantly less time climbing the bars than male wild type mice (Fig. 6b). Furthermore, when comparing the average bar climbing time between wild type and knockout mice regardless of sex, Nestin-Cre-Lpd KO mice showed a significantly lower bar climbing time than wild type mice (Fig. 6c).

No significant effects of either genotype or sex were identified for the rest of the variables recorded during the test: latency to object displacement, number of exploration episodes and number of bar climbing episodes.

### Rotarod test

**Figure 7:**
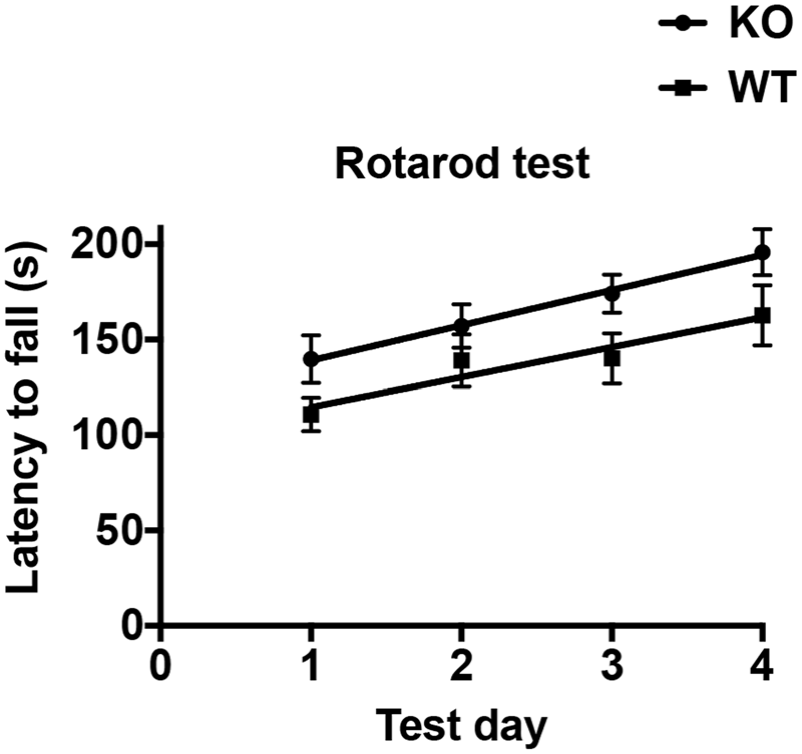
Nestin-Cre-Lpd KO mice showed higher basal levels of motor coordination in the rotarod test. Average latency to fall (seconds) from the rotarod apparatus during four consecutive trial sessions (Mean ± SEM). A linear regression of Two-way ANOVA of the data obtained from the rotarod trials yielded a linear slope of WT: 15.67 ± 5.81, SEM, n = 15, vs KO: 18.46 ± 5.13, SEM, n = 20 with no significant difference between the linear slopes (F[1,136] = 0.13, p = 0.7207), but the X intercept value of WT: 98.96 ± 15.92 s, SEM, vs KO: 120.5 ± 14.03 s, SEM was significantly different (F[1,137] = 10.85, p = 0.0013, Two-way ANOVA) suggesting an enhanced basal level of motor coordination.

Both genotypes showed the expected improvement in the ability to stay on top of the rotating cylinder during successive trial sessions. A linear regression of the data obtained from the rotarod trials showed no significant difference between the linear slopes but the X intercept value for the curve corresponding to Nestin-Cre-Lpd KO mice was significantly higher than the one for the wild type curve (Fig. 7). This indicates a basal difference in the latency to fall off the apparatus with the Nestin-Cre-Lpd KO mice displaying greater motor co-ordination compared to their wild type littermates but no differences in motor learning with all groups showing learning over the four test days.

## Discussion

Our results show that brain specific conditional Lpd knockout mice exhibit an abnormal behavioural pattern of hyperactivity and anxiety that is consistently evident across a number of different behavioural tests.

SHIRPA designates a standardized battery of behavioural tests designed to provide a quick and sensitive assessment of a wide number of behavioural parameters^22^. The performance of Nestin-Cre-Lpd KO mice during the test suggests that sensory abilities and basic reflexes appear to be intact. However, a subset of the quantified variables showed significant differences according to the genotype of the individual. The levels of spontaneous activity displayed when they were under observation in the viewing jar were unusually high in Nestin-Cre-Lpd KO mice. Furthermore, the tendency of the Nestin-Cre-Lpd KO mice to explore when transferred to a novel arena, both in terms of locomotion across the novel environment and the frequency of rearing behaviour, was also elevated when compared to their wild type littermates. Taken together, these results are suggestive of a hyperactive phenotype in the knockout animals manifested in both sexes, while at the same time they rule out the presence of basic sensory or neurological impairments derived from the absence of Lpd.

The light/dark box test is normally used for the assessment of anxiety levels, and time spent in the lit half of the arena is considered to be the most reliable parameter to estimate them. In the present study, there were no significant differences for this variable between knockout and wild type mice. However, Nestin-Cre-Lpd KO mice showed enhanced levels of exploration while in the darkened chamber, expressed as a significantly longer distance covered. This is consistent with the increased exploratory behaviour displayed during the SHIRPA test.

In the open field test the time the male Nestin-Cre-Lpd KO mice spent exploring the inner zone was significantly lower than that of their male wild type littermates. Since animals that spend less time in the inner zone of the open field arena are normally considered to be more anxious compared to those that prefer the perimeter adjacent to the walls^23^, these results suggest the existence of higher levels of anxiety in male Lpd KO animals. Independent of sex, we observed for all Nestin-Cre-Lpd KO mice that latency to enter this area in the first place was noticeably longer, further suggesting increased anxiety. However, this contradicts the results from the light/dark box test, which suggested that anxiety levels were no different than in wild type animals. Since the outcome of the open field test is known to show important variations according to the particular experimental conditions^24^, it is possible that our chosen conditions were stringent enough to uncover a slight increase in anxiety levels in the male Lpd KO mice that is not evident when probed by the smaller, less anxiety-generating light/dark box. The open field test likely reflects both anxiety levels versus exploratory behaviour of the mouse, given exploration of this arena represent a conflict between the drive to explore versus avoidance of a potentially threatening area^25^. It is interesting to note that, in agreement with what was observed in previous tests, Nestin-Cre-Lpd KO mice also showed signs of hyperactivity in the open field. Their speed within the inner zone was significantly higher than that of their wild type littermates and the latency to show rearing behaviour in the inner zone was significantly lower. In addition, there were clear trends that almost reached significance for a lower latency to show rearing behaviour in the outer zone and the frequency of jumping behaviour during the test.

There were also differences when the mice were confronted with a novel object, but interestingly they were highly dependent on the sex of the individual. Nestin-Cre-Lpd KO females spent more time than wild type females investigating the novel object but there were no differences between the males. Independent of sex, we observed during this novel object exploration test that all Nestin-Cre-Lpd KO mice also spent less time climbing the cage bars. Since bar climbing was in this context a proxy of cage exploration as opposed to novel object exploration, this suggests reduced exploration of cage environment by Nestin-Cre-Lpd KO mice.

The abnormal phenotype of the Nestin-Cre-Lpd KO mice was for some specific variables only evident in one of the sexes. This is not entirely surprising, since important sex differences in behavioural tests in rodents have been identified for decades, and this is especially true for those tests aimed at measuring anxiety levels^26^. It may be the case that specific characteristics of the CNS in one of the sexes obscures the effect of the absence of Lpd, and therefore this absence may become evident in the behavioural phenotype of individuals of the opposite sex only. Our results highlight the importance of including experimental subjects of both sexes in any attempt to characterize the behavioural phenotype of any given mouse model in order to minimize the risk of missing some potentially meaningful differences because of this fact^27^.

Both wild type and Nestin-Cre-Lpd KO mice showed the expected improvement in their ability to stay on top of the rotating cylinder during successive trial sessions in the rotarod (motor learning), as evidenced by the good fit of the data to a linear curve with slopes that were significantly different from zero but no different from each other. However, the difference in the X intercept values between the two curves indicates that during any single trial during the testing protocol the Nestin-Cre-Lpd KO mice took longer to fall off the apparatus and therefore exhibited a better motor coordination compared to wild type littermates. This in turn suggests that the absence of Lpd during CNS development does not impair motor learning per se, but it does seem to have an improving effect of the basal levels of the type of motor coordination that the animal is capable of displaying in the rotarod, and this difference is consistently maintained throughout training. This outcome is also in agreement with the enhanced mobility and speed that KO animals showed in the other tests in this study.

Remarkably, we failed to find any difference between the genotypes for the variables quantified during home cage monitoring. We conclude that the signs of hyperactivity and enhanced exploratory behaviour that were evident in the other test must require the mouse to be introduced into a novel environment (the testing apparatus) in order to become evident. In the home cage, where anxiety levels and tendency to explore can be expected to be at minimum due to prolonged habituation, KO individuals appear to adopt a behavioural pattern that is indistinguishable from that of their wild type littermates.

Taken together, our behavioural analysis of CNS specific Lpd knockout mice revealed hyperactivity and increased anxiety. On the cellular level these phenotypes may be due to Lpd’s known role in axonal and dendritic extension/branching and neuronal migration, which may cause altered neuronal connectivity during development. Since Lpd’s *C*. *elegans* orthologue MIG-10 supports synaptic vesicle clustering^18^ and Lpd promotes endocytosis^6^ and localises to synapses (M. Krause, unpublished observation), loss of Lpd may affect synapse function and/or dendritic spine formation and dynamics and we are exploring these various possibilities. To our knowledge, this set of results represent the first piece of evidence suggesting that the previously characterized role of Lpd in the regulation of neuronal morphogenesis and migration has a behavioural correlate in mammals. Further studies will elucidate the specific subsystems within the CNS affected by the absence of Lpd that are responsible for the observed phenotype.

## Materials and Methods

### Western blot experiments

Meninges were removed from mouse brains of adult Nestin-Cre-Lpd KO mice and Lpd flox/flox controls and cortices were dissected. Cortices were lysed in lysis buffer (50 mM Tris HCL; 200 mM NaCl; 1% NP-40; 2 mM MgCl_2_; 10% Glycerol (pH 7.4); 1 mM Na_3_VO_4_; 10 mM NaF; protease inhibitors (complete mini without EDTA, Roche) with a 10 second burst of a Polytron (PT1200E) blender. Lysates were incubated on ice for 30 min, centrifuged at 17,000 × g at 4 °C for 15 min and protein concentration determined (Pierce® BCA protein assay kit (Thermo Fisher). Equal amounts of lysates (30 µg) were separated on SDS-PAGE gels, transferred onto Immobilon-P membranes (Millipore), blocked in 10% fetal calf serum and probed with the indicated antibodies, followed by HRP-secondary antibodies (DAKO). Blots were developed with the Immun-Star WesternC™ ECL kit using the Biorad Imager and ImageLab software.

### Antibodies

Lpd rabbit antiserum 3917^1^; VASP rabbit mab 9A2 (3132, Cell Signalling), Mena mouse mab A351F7D9^28^, HSC70 mouse mab (Santa Cruz), Scar1 mouse mab (BD-Transduction Labs), Scar/WAVE2 rabbit mab D2C8 (3659, Cell Signalling). Secondary antibodies: HRP-goat anti-rabbit, goat anti-mouse (Dako).

### Animal experimental procedures

All experimental procedures in this study using mice were approved by the local ethical review panel (ERP) of King’s College London, and the United Kingdom Home Office (license PPL: 70/7184) according to the UK Home Office Animals Scientific Procedures Act 1986. All efforts were made to minimize animal suffering and to reduce the number of animals used.

### Conditional knockout Lpd mice

The generation of the C57BL/6 conditional Lpd (RAPH1) knockout mice has been described previously^7^ and these mice were crossed with C57BL/6 mice heterozygous for Nestin-Cre (kindly provided by Axel Behrens, CRICK, London, UK) to generate Nestin-Cre-Lpd KO mice on a C57BL/6 genetic background. Nestin-Cre-Lpd KO (Lpd flox/flox; Nestin-Cre/-) mice (heterozygous for Nestin-Cre) and their wild type (Lpd flox/flox) littermates of both sexes were used as experimental subjects. Mice were housed in standard cages measuring 32 × 16 × 14 cm with sawdust (Litaspen premium, Datesand Ltd, Manchester), a cardboard shelter and additional bedding material (Sizzlenest, Datesand Ltd, Manchester) with ad libitum access to water and food (Rat and Mouse 3 Diet, Special Diet Services, Essex, UK). The housing and test rooms were maintained at constant room temperature (21 °C) and humidity (45%) and kept under a regular light/dark schedule with lights on from 07:30 to 19:30 hours (light = 270 lux). All animals were tested when they were between three and six months of age, and were individually housed one week before the start of the testing protocol. Animals were singly housed when adult to avoid any potential confounds from social hierarchies and aggressive behaviour hierarchies, which could influence the controlled assessment of social behaviours^29^. The oestrous phase of the female mice was not checked in this study. However, it is unlikely that this affected results because there were no major effects in the variance between males and females. Sawdust was changed every other week but never on the day before or the day of testing and the enrichment (nesting material and house) was changed less regularly to minimize the disruption to the animals. Numbers (n = 8–12/genotype/sex) were based on typical sample sizes used in behavioural testing. To detect differences in behaviour, sample sizes of 8–12 animals of each sex per group should be sufficient to obtain statistical significance, based on power analysis using a significance level of p < 0.05 with a 2-sided test, a power of 90%, and moderate effect size (Cohen’s d = 1.4). The effect size was calculated from our published data on the C57BL/6 J background strain^30^ and the recommendation of the Mouse Phenome Project (http://phenome.jax.org/). The same cohort of mice was used for all tests. All experiments, with the exception of the home cage monitoring, were performed during the daytime light on phase (2 PM−5 PM) of the regular light/dark schedule. The control or Nestin-Cre-Lpd KO mice were tested in a random order. The sequence of the tests was: SHIRPA, homecage, open field, light/dark test, novel object, and rotarod. The tests were spaced so that there was at least a full week between one test and the next.

### SHIRPA procedure

A shortened version of the SHIRPA behavioural tests adapted from Rogers *et al*.^22^ was used for primary assessment of the behavioural profile exhibited by Nestin-Cre-Lpd KO (Lpd flox/flox; Nestin-Cre/-) mice and their wild type (Lpd flox/flox) littermates. The protocol began with the observation of undisturbed behaviour in a cylindrical viewing jar (15 × 12.5 cm), followed by transfer to an arena (35 w × 50 l × 18 h cm divided into fifteen 10 × 10 cm squares), a series of tail manipulations and use of a grid to measure grip strength and righting reflex. The following variables were recorded during the protocol: body position, spontaneous activity, respiration rate, presence of tremor, urination, transfer arousal, palpebral closing, piloerection, gait, pelvic elevation, tail elevation, startle response, escape response to touch, tail suspension (trunk curl, limb grasp and visual placing), grip strength, wire manoeuvre, righting reflex, negative geotaxis, fear, irritability, aggression and vocalization. For each variable, a range was defined and a score was assigned to each mouse according to its performance by a single observer who remained blind to their genotype. In addition, the number of faecal boli, the number of rearing events in the arena, and general locomotor activity (defined as number of squares entered by all four paws in 30 seconds) were recorded during the protocol.

### Light/Dark box

The experimental apparatus consisted of a white acrylic box (44 × 21 × 21 cm) unequally divided into two chambers by a 21 × 50 cm acrylic separator with a small opening (5 × 7 cm) that allows the mouse to switch freely between the two chambers. The larger chamber was brightly lit (100 lux) using an overhead lamp, while the smaller chamber was dimly illuminated (15 lux). The experimental protocol was based on Bourin & Hascoët (2003)^31^. Mice were placed in the dark compartment and their movement recorded over five minutes using a camera positioned above the apparatus. Mean velocity (cm per second) and total distance (cm) covered in each compartment were extracted from the recordings using the EthoVision tracking software (EthoVision, Noldus Information Technologies, Wageningen, The Netherlands). In addition, the latency to enter, the number of entries into (defined as all four paws in one compartment) and the time spent in, each compartment were manually scored by an observer who was blind to the sex and genotype of each mouse. If a mouse failed to enter the light chamber during the duration of the experiment, its latency was defined as 300 s.

### Open field

The open field test was carried out using a square white acrylic arena (40 × 40 × 40 cm) evenly lit from below by artificial light (20 lux). Mice were placed in the same start location in the outer zone of the open field, facing an outer wall of the arena and allowed to explore it for ten minutes. Their movements and location throughout the duration of the test were recorded using a camera positioned overhead. The recordings were analysed with EthoVision. A square “inner” zone measuring 20 × 20 cm was virtually drawn in the centre of the open field by the tracking software, with the remaining boundary area adjacent to the arena walls defined as the “outer” zone. Variables extracted by the software from the test recordings included distance and speed travelled across the outer and inner zones; total entries and total time spent in each zone and latency to enter the inner zone. In addition, rearing and jumping in the outer and inner zone were manually scored by an experienced behavioural researcher who was blind to the genotype of the mice.

### Home cage monitoring

The experimental protocol was adapted from Mill *et al*.^32^. Locomotor activity was recorded for two hours in the middle of the dark phase of the light/dark cycle using a camera positioned above the test arenas, with the testing room illuminated by six red multi-LED cluster lamps (No. 310-6757; RS Components Northants, UK of approximate wavelength 705 nm) which provided minimal light for recording during the dark phase. Mice were filmed in their home cage with the cage lid replaced by a clear acrylic lid containing breathing holes. Petri dishes containing wetted food pellets were placed in each cage to provide food and water during the recording period, and the mice were habituated to the dishes for three consecutive days before being filmed. The recordings were analysed using EthoVision, from which the following variables were derived: total distance moved, average speed, heading, turn angle, angular velocity and meander.

### Novel object exploration

The tests were performed in a clean home cage with a standard amount of sawdust bedding and a regular cage lid, without food pellets or water bottle. Low red light illumination was provided with multi-LED cluster lamps to allow for effective capture of movement during the test. Activity was recorded with a camera in a lateral position to the cage. Mice were habituated to the cage for 20 minutes, after which an unfamiliar object (a rectangular wooden bar, 1 cm × 1 cm × 4.5 cm) was introduced into one end of the cage. The behaviour was recorded for 10 minutes. The following variables were manually scored from the recordings by an observer who was blind to the sex and genotype of each mouse: latency to object displacement, total object exploration time, number of exploration episodes (defined as direct contact between the muzzle and the object), total cage lid bar climbing time and number of cage lid bar climbing episodes.

### Accelerating rotarod test

The test was performed using an Ugo Basile 7650 accelerating rotarod apparatus (Linton Instruments, Diss, UK) programmed to accelerate from 4 rpm to 40 rpm over a period of 300 s. Each mouse was placed on the rotating drum and allowed to habituate for 20 seconds at the baseline speed before starting the acceleration protocol. The variable recorded was the time (in seconds) it took for each mouse to fall off the rotating barrel after initiation of the protocol. If a mouse failed to fall off at the end of the test, its latency was recorded as 300 s. Each session took place during the light phase of the cycle and consisted of three individual trials. The mice underwent a total of four test sessions during four consecutive days.

### Statistical analysis

Two-way ANOVA with genotype and sex as the independent factors was used to assess the statistical significance of mean differences obtained from the raw data. In those cases in which a significant interaction between the two factors was observed, or both factors had a significant effect on the variable independent from each other, separate analyses of each factor were performed using unpaired, two tailed Student’s t-tests. Chi-square tests were used for assessing significant differences between proportions. Data from the rotarod test was analysed by performing a non-linear regression to determine the optimal curve fit followed by a standard linear regression. For an assessment of the normal distribution of the data both the D’Agostino-Pearson and Shapiro-Wilk test were taken into account and the Grubbs method was used to identify outliers. All the calculations were performed using the PRISM 7 statistical package (GraphPad Software Inc., La Jolla, CA).

### Data availability

The datasets generated during and/or analysed during the current study are available from the corresponding author on reasonable request.
